# Sparse Linear Surrogates Match Neural Network Potentials on the SPICE Biomolecular Benchmark with Three Orders of Magnitude Smaller Training Sets

**DOI:** 10.1021/acs.jpclett.6c01491

**Published:** 2026-07-06

**Authors:** D. L. Azevedo

**Affiliations:** Institute of Physics, University of Brasília (UnB), Campus Universitário Darcy Ribeiro, Asa Norte, 70919-970 Brasília, Distrito Federal, Brazil

## Abstract

We introduce the orbital cluster expansion (OCE), a linear regression on physics-motivated local features derived from atomic orbital eigenenergies, and benchmark it against the SPICE 2.0 biomolecular data set at the ωB97M-D3BJ/def2-TZVPPD level. With regression of formation energies on 677 dipeptides spanning the natural amino acids, ridge regression on 414 OCE features attains a parent-stratified test root-mean-square error of 30 meV per atom with Spearman ρ = 0.97 and *R* ^2^ = 0.95 against a target spread of only 0.13 eV per atom, matching MACE-OFF23­(L) and ANI-2x trained with 10^4^–10^6^ conformations but with ∼10^3^ fewer training points. Comparable accuracy holds on 500 PubChem drug-like molecules and 500 DES370K dimers. We characterize a fundamental dual regime: intermolecular ranking is preserved across chemistries, while intraconformer ranking is random because the basis cannot resolve geometry-only variation within a fixed connectivity. OCE is a transparent, physically interpretable surrogate for intermolecular biomolecular screening.

## Graphical abstract

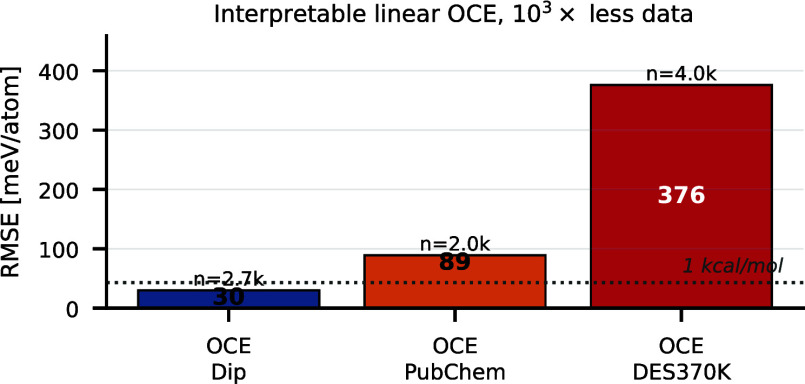

Quantum-mechanical reference data have driven a generation of machine learning interatomic potentials that approach density functional theory (DFT) accuracy at orders-of-magnitude lower cost.^1–5^ The leading neural network potentials for biomolecular systems, ANI-2x,^6^ SchNet,^3^ and MACE-OFF,^7^ consume training sets of 10^4^–10^7^ conformations computed at hybrid-DFT or near-coupled-cluster levels, and the most recent general purpose models (e.g., UMA and MACE-OMOL) push both data set size and chemical coverage further still. Beyond the substantial cost of generating these references, the resulting models are notoriously opaque, which hampers physical interpretation and complicates transfer between chemistries.

The cluster expansion (CE) of Sanchez and Ducastelle,^8^ later formalized for alloy thermodynamics by the UNCLE algorithm,^9^ provides a complementary paradigm: a strictly linear regression on products of site-occupation variables, with explicit symmetry handling and a small, interpretable basis. The CE has remained restricted to periodic substitutional alloys because its configurational variable σ_*i*_ ∈ {−1, +1} encodes only “which species at site *i*”, with no notion of bond connectivity, geometry, or molecular topology.

Here, we extend the CE concept to molecules through an orbital cluster expansion whose configurational variable is the atomic orbital eigenenergy ε_μ_ obtained from a single semi-empirical calculation per element.^10^ The basis is hierarchical (Figure 1)­

1 $$\Pi_{F}^{1F}=\underset{a\in F}{\sum }n_{a}\varepsilon_{\mu \left(a\right)}$$

2 $$\Pi_{F}^{2F}=\underset{\left(a,b\right)\in F}{\sum }\kappa_{ab}\lambda_{ab}^{-}\left(r_{ab}\right)$$

3 $$\Pi_{F}^{3F}=\underset{\left(i,j,k\right)\in F}{\sum }\cos\left(\theta_{ijk}\right){\mathrm{\varepsilon ̅}}_{ijk}$$

4 $$\Pi_{F}^{4F}=\underset{\left(i,j,k,l\right)\in F}{\sum }\cos\left(2\phi_{ijkl}\right){\mathrm{\varepsilon ̅}}_{ijkl}$$

where the index *F* labels a symmetry-distinct local cluster, a figure in the cluster expansion sense: a single atom (1*F*), a bonded pair (2*F*), an angular triple (3*F*), or a dihedral quadruplet (4*F*). Π_*F*_ is the correlation function for figure *F*, summed over every instance of that figure in the molecule, and *J*_*F*_ (below) is its fitted coefficient. Here, *n*_*a*_ is the orbital occupation; κ_*ab*_ is the bond order; λ_*ab*_^–^ is the bonding eigenvalue of the 2 × 2 linear combination of atomic orbitals (LCAO) secular problem with off-diagonal hopping *h*(*r*) = *h*_0_ exp­[−α­(*r* – *r*_0_)];^11^ θ_*ijk*_ is the bond angle; ϕ_*ijkl*_ is the dihedral angle; and ε̅ is the geometric mean of orbital energies along the figure. The Π^3*F*^ figure key is augmented by the smallest ring size containing the angle. The total energy is expanded as *E*_OCE_ = ∑_*F*_ *J*_*F*_Π_*F*_, and the coefficients {*J*_*F*_} are obtained by ridge regression on per-atom features and per-atom DFT energies.

**Figure 1:**
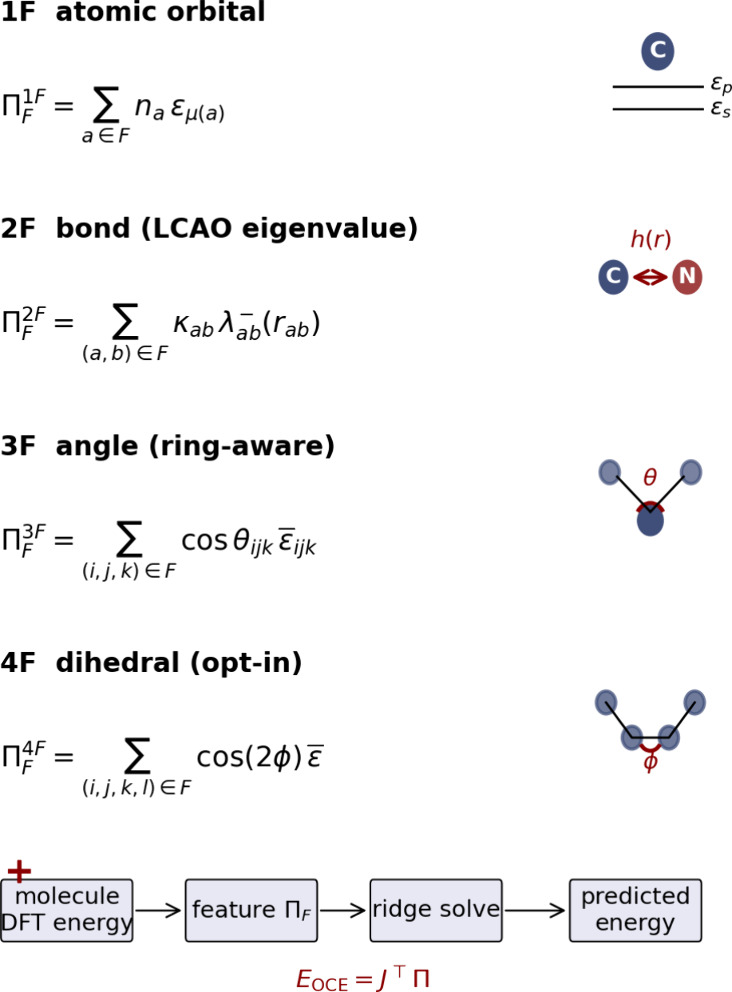
Hierarchical OCE basis. 1*F* sums atomic orbital eigenenergies. 2*F* accumulates the bonding eigenvalue of a 2 × 2 LCAO secular problem with distance-dependent hopping *h*(*r*). 3*F* carries an angular factor cos θ and a ring size key. 4*F* adds an optional dihedral-resolved basis. The whole pipeline reduces to a single ridge solve once the figure keys are enumerated.

We benchmark OCE on three subsets of SPICE 2.0:^12^ the 677 zwitterion-blocked dipeptides at the ωB97M-D3BJ/def2-TZVPPD level^13^ (5 conformations per molecule), 500 PubChem-Set-1 drug-like molecules (5 conformations each, including elements through Br and I), and 500 DES370K^14^ noncovalent dimers, including ion pairs and π-stacked aromatics (10 conformations each). OCE is trained and evaluated independently within each subset; the only quantities shared across subsets are the single-atom orbital reference energies, which are element constants rather than training data. Holdout splits are made by parent molecule, the distinct chemical species (fixed connectivity) that the SPICE conformers sample: every conformation of a given parent goes either entirely to training or entirely to the held-out test set. This is a strict generalization test that excludes the trivial mode of interpolating between conformers of a known parent.

Throughout, we regress formation energies, the per-atom DFT energy with the element-dependent atomic self-energies removed, which is the chemically meaningful target: total energies are dominated by those self-energies; therefore, ranking molecules by total energy is a near-trivial composition count and not a meaningful measure of model quality. On dipeptide formation energies, whose entire held-out test spread is only 0.13 eV per atom, the ridge model attains a root-mean-square error of 30 meV per atom, Spearman ρ = 0.97, and *R* ^2^ = 0.95 (Figure 2A). The fact that a linear model resolves 95% of the variance of a target this narrow shows that it captures genuine intensive energetics and not composition. The intermolecular RMSE is 30 meV per atom on dipeptides, 89 meV per atom on PubChem drug-like molecules, and 376 meV per atom on DES370K dimers, with the variation scaling with the chemical diversity of each subset (5, 10, and 15 distinct elements, respectively) and with the prevalence of long-range electrostatics. Evaluated on our identical molecule-holdout split (section S5 of the Supporting Information), MACE-OFF23 ^7^ attains an energy RMSE of the same order while resolving the intraconformer ordering that OCE cannot (within-parent ρ ≈ 0.96 versus 0.09); both methods are limited by the net-charged residues. OCE reaches this regime with 2.7 × 10^3^ training conformations, roughly 10^3^ fewer than the neural network potentials,^6,7^ using a 414-feature basis and a single ridge solve.

**Figure 2:**
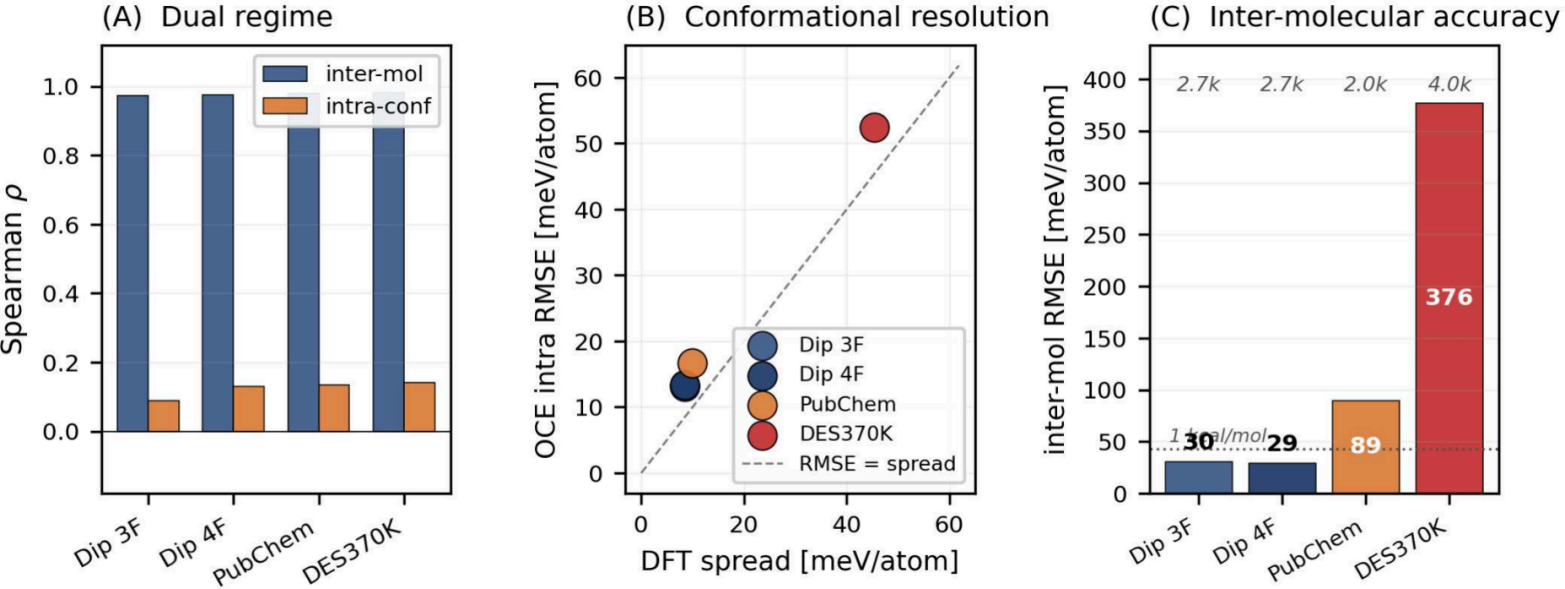
Dual-regime nature of the OCE basis on SPICE 2.0 (formation energies). (A) Spearman rank correlation between predicted and DFT energies on parent-stratified test sets, separated by intermolecular (blue) and within-parent intraconformer (orange) ranking. The intermolecular regime is recovered (ρ ≳ 0.97) across all chemistries; the intraconformer regime collapses to noise. (B) Within-parent root-mean-square error versus within-parent DFT spread; the diagonal indicates the boundary above which the model predicts noise larger than the true conformational variation. (C) Intermolecular formation energy RMSE for each subset; italic numbers above each bar give the training set size and the dotted line marks chemical accuracy (1 kcal/mol ≈ 43 meV per atom). A controlled comparison to MACE-OFF23 on the identical test set is given in section S5 of the Supporting Information.

The basis is also strictly interpretable, and because the model is fitted to formation energies, its coefficients carry genuine chemical meaning rather than tracking composition. Ranked by standardized magnitude, the leading *J*_*F*_ coefficients are the 2*F* bonding eigenvalues of the bonds that define peptide energetics, the amide C–N, carbonyl C=O, C–H, and C–C bonds, consistent with the formation energy being governed by bond formation (the full ranking is tabulated in section S1 of the Supporting Information). An analysis of worst fit dipeptide parents (section S3 of the Supporting Information) shows that residual errors concentrate on amino acid pairs with strong inter-residue electrostatics (Asp–Arg, Arg–Cyx, Asp–Hip, Lys–Glu, and Hip–Pro), where local features underdescribe the long-range Coulomb interaction.

We probe the within-parent axis explicitly: across each held-out parent’s own conformers the mean Spearman correlation collapses to ∼0.08 (dipeptides), 0.13 (PubChem), and 0.14 (DES370K) (Figure 2A; full distribution in Figure 3) and is unchanged by the choice of total or formation target. Within a parent, the composition is fixed; therefore, the two differ only by a constant and rank identically. Doubling the basis to 886 features with the dihedral four-figure Π^4*F*^ does not help. The basis is therefore conformationally blind: conformers share the same connectivity, giving near-identical figure totals and a near-constant per-parent prediction.

**Figure 3:**
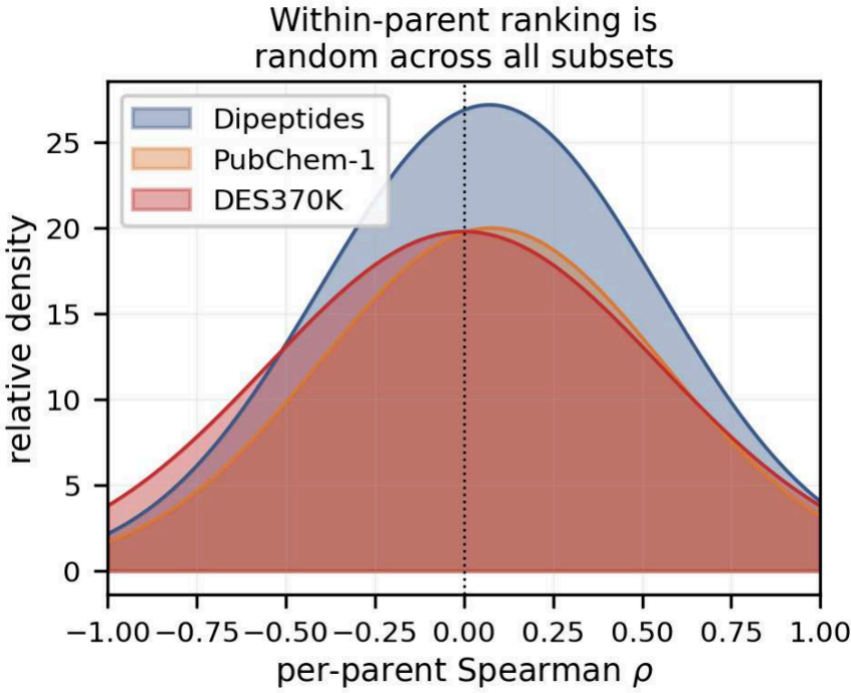
Distribution of the within-parent Spearman correlation between OCE-predicted and DFT energies, evaluated independently for each parent molecule of the held-out test set across the three SPICE 2.0 subsets. All three distributions are centered near zero: the conformational substructure is invisible to the local basis even where absolute per-atom prediction is accurate. The distribution is identical for total and formation energy targets since, within one parent, the composition (hence, the per-parent offset) is fixed.

This dual-regime behavior follows from the structural design of the basis. The 1*F*, 2*F*, and 3*F* figures are summed over local environments defined by graph adjacency; therefore, two conformers with identical adjacency graphs differ only in bond lengths and angles, variations that enter only through the smooth functions λ_*ab*_^–^(*r*) and cos θ, both of which are shallow within a conformer well. Capturing the residual conformational variation requires either a neighborhood density expansion as in the atomic cluster expansion^4^ and moment tensor potentials^15^ or explicit noncovalent contact features in 2*F*.

The dual regime has a sharp practical interpretation. Intermolecular ranking, where OCE excels, is the central task in constitutional and fragment-based screening, whereas a conformer search of a fixed molecule, where OCE fails, remains the domain of geometry-resolving neural network potentials (MACE^5^ and NequIP^16^); OCE also transfers to molecule classes absent from training when these span the figure basis (section S6 of the Supporting Information). Where large pretrained models already exist, the value of OCE is therefore not raw data efficiency but transparency: every feature has a closed form physical meaning; training is a single subsecond ridge solve; and the model is directly compatible with the combinatorial selection and ground-state-priority-weighting (Garbulsky–Ceder)^17^ machinery of alloy CEs, using standard tools (ASE^18^ and scikit-learn^19^).

In summary, the orbital cluster expansion attains MACE-OFF-level intermolecular accuracy on three SPICE subsets (on formation energies, the meaningful target), with 3 orders of magnitude fewer training conformations, while being intrinsically blind to within-conformer geometry by construction. Two linear-regression-preserving extensions follow: (i) screened Coulomb and van der Waals two figures for noncovalent and electrostatic contacts (a single formal charge Coulomb feature already improves the charged DES370K dimers; section S7 of the Supporting Information) and (ii) higher order figures (5*F*/6*F*) resolving conformational substructure, both to be reported in companion work.

## Supplementary Material



## Notes

Detailed methodology (basis derivation, ridge/Garbulsky–Ceder/simulated annealing implementations, and atomic orbital reference table), total versus formation energy comparison for all three subsets, per-parent error tables, four-figure ablation, head-to-head evaluation of MACE-OFF23 (S/M/L) on the identical held-out dipeptide test set, cross-subset transferability study, long-range Coulomb/Madelung feature ablation, hyperparameter sensitivity, SPICE 2.0.1 download and feature cache instructions, and computational environment (PDF)

## References

[1] Behler J., Parrinello M. (2007). Generalized neural-network representation of high-dimensional
potential-energy surfaces. Phys. Rev. Lett..

[2] Smith J. S., Isayev O., Roitberg A. E. (2017). ANI-1:
an extensible neural network
potential with DFT accuracy at force field computational cost. Chemical Science.

[3] Schütt K. T., Sauceda H. E., Kindermans P.-J., Tkatchenko A., Müller K.-R. (2018). SchNet—A deep learning architecture for molecules
and materials. J. Chem. Phys..

[4] Drautz R. (2019). Atomic cluster
expansion for accurate and transferable interatomic potentials. Phys. Rev. B.

[5] Batatia, I. ; Kovács, D. P. ; Simm, G. N. C. ; Ortner, C. ; Csányi, G. MACE: higher order equivariant message passing neural networks for fast and accurate force fields. Proceedings of the Advances in Neural Information Processing Systems; New Orleans, LA, Nov 28–Dec 9, 2022; Advances in Neural Information Processing Systems 35, pp 11423–11436, 10.52202/068431-0830.

[6] Devereux C., Smith J. S., Huddleston K. K., Barros K., Zubatyuk R., Isayev O., Roitberg A. E. (2020). Extending
the applicability of the
ANI deep-learning molecular potential to sulfur and halogens. J. Chem. Theory Comput..

[7] Kovács D. P., Moore J. H., Browning N. J., Batatia I., Horton J. T., Pu Y., Kapil V., Witt W. C., Magdău I.-B., Cole D. J., Csányi G. (2025). MACE-OFF:
short-range transferable
machine learning force fields for organic molecules. J. Am. Chem. Soc..

[8] Sanchez J. M., Ducastelle F., Gratias D. (1984). Generalized cluster description of
multicomponent systems. Physica A: Statistical
Mechanics and its Applications.

[9] Lerch D., Wieckhorst O., Hart G. L. W., Forcade R. W., Müller S. (2009). UNCLE: a code
for constructing cluster expansions for arbitrary lattices with minimal
user-input. Modell. Simul. Mater. Sci. Eng..

[10] Bannwarth C., Ehlert S., Grimme S. (2019). GFN2-xTB:
an accurate and broadly
parametrized self-consistent tight-binding quantum chemical method
with multipole electrostatics and density-dependent dispersion contributions. J. Chem. Theory Comput..

[11] Wolfsberg M., Helmholz L. (1952). The spectra and electronic structure
of the tetrahedral
ions MnO_4_
^–^, CrO_4_
^2–^, and ClO_4_
^–^. J.
Chem. Phys..

[12] Eastman P., Pritchard B. P., Chodera J. D., Markland T. E. (2024). Nutmeg and SPICE:
models and data for biomolecular machine learning. J. Chem. Theory Comput..

[13] Mardirossian N., Head-Gordon M. (2016). *ω*B97M-V: a
combinatorially optimized,
range-separated hybrid, meta-GGA density functional with VV10 nonlocal
correlation. J. Chem. Phys..

[14] Donchev A. G., Taube A. G., Decolvenaere E., Hargus C., McGibbon R. T., Law K.-H., Gregersen B. A., Li J.-L., Palmo K., Siva K. (2021). Quantum chemical benchmark databases of gold-standard
dimer interaction energies. Scientific Data.

[15] Shapeev A. V. (2016). Moment
tensor potentials: a class of systematically improvable interatomic
potentials. Multiscale Modeling & Simulation.

[16] Batzner S., Musaelian A., Sun L., Geiger M., Mailoa J. P., Kornbluth M., Molinari N., Smidt T. E., Kozinsky B. (2022). E­(3)-equivariant
graph neural networks for data-efficient and accurate interatomic
potentials. Nat. Commun..

[17] Garbulsky G. D., Ceder G. (1995). Linear-programming method for obtaining
effective cluster interactions
in alloys from total-energy calculations: application to the fcc Pd-V
system. Phys. Rev. B.

[18] Larsen A. H., Mortensen J. J., Blomqvist J., Castelli I. E., Christensen R., Dułak M., Friis J., Groves M. N., Hammer B., Hargus C. (2017). The atomic simulation environment—A Python library
for working with atoms. J. Phys.: Condens. Matter.

[19] Pedregosa F., Varoquaux G., Gramfort A., Michel V., Thirion B., Grisel O., Blondel M., Prettenhofer P., Weiss R., Dubourg V. (2011). Scikit-learn: machine
learning in Python. Journal of Machine Learning
Research.

