# Supplementary material for: Sparse Linear Surrogates Match Neural Network Potentials on the SPICE Biomolecular Benchmark with Three Orders of Magnitude Smaller Training Sets
Source: J Phys Chem Lett. 2026 Jul 6;17(28):7867–70. doi: 10.1021/acs.jpclett.6c01491 (PMC13383835; doi:10.1021/acs.jpclett.6c01491)
Supplement: Supplementary file 1 [file jz6c01491_si_001.pdf]

Supporting Information for: Sparse Linear Surrogates Match Neural-Network Potentials on the SPICE Biomolecular Benchmark with Three Orders of Magnitude Smaller Training Sets

D. L. Azevedo*,†,‡

†*Institute of Physics, University of Brasília (UnB), Campus Universitário Darcy Ribeiro – Asa Norte, 70919-970, Brasília, DF, Brazil*

‡*ORCID: 0000-0002-3456-554X*

E-mail: david888azv@unb.br

1 Detailed methodology

1.1 Atomic orbital reference table

For each chemical element E present in the dataset (H, C, N, O, F, S, P, Cl, Br, I), a single-atom GFN2-xTB calculation[1] is performed in vacuum at zero charge and ground-state unrestricted-Hartree-Fock multiplicity. Frontier orbital eigenenergies are extracted, grouped by shell label (s, p, d) according to degeneracy, and stored as (label, ε, occupation) tuples. The reference is shared across all OCE training and prediction calls and regenerated only when a new element is encountered. Orbital eigenenergies are converted to electronvolts using the Hartree-to-eV factor 27.211 386 eV/Ha.

1.2 Bond perception

Bonds are detected from interatomic distances using elemental covalent radii[2] with a multiplicative cutoff of 1.30. For each candidate pair (a, b) the distance ratio $\rho_{ab} = r_{ab}/(r_a^{\mathrm{cov}} + r_b^{\mathrm{cov}})$ classifies the integer bond order κ_{ab}:

ρ_{ab} range	κ_{ab}
[0.00, 0.83)	3 (triple)
[0.83, 0.93)	2 (double)
[0.93, 1.30)	1 (single)

A valence-correction heuristic prevents over-bonding: for each atom we compare the running sum of accepted bond orders against the typical valence (C 4, N 3, O 2, S 2, ...); if a candidate would exceed the budget, its order is downgraded to single, and if that still exceeds, the bond is rejected.

1.3 Figure enumeration

After bond perception the $1F$ figures are atomic (element, shell) pairs; $2F$ figures are bonded (atom$_a$, atom$_b$, bond order) triples; $3F$ figures are angular triples (centre, arm 1, arm 2) where both arms are bonded to the centre. The smallest ring (4–10) that contains the angle is appended to the figure key by a breadth-first traversal of the bond graph. For periodic cells, $3 \times 3 \times 1$ supercell replication is applied before ring detection to remove false short-cycle artefacts introduced by periodic wrap-around.

1.4 Correlation functions

For each unique figure key, we sum the corresponding Π_F values over all instances of that figure in the molecule:

$$\Pi_F^{1F} = \sum_{a \in F} n_a \, \varepsilon_{\mu(a)}, \tag{1}$$

$$\Pi_F^{2F} = \sum_{(a,b) \in F} \kappa_{ab} \, \frac{1}{2} \left[(\varepsilon_a + \varepsilon_b) - \sqrt{(\varepsilon_a - \varepsilon_b)^2 + 4h(r_{ab})^2} \right], \tag{2}$$

$$h(r) = h_0 \exp[-\alpha(r - r_0)], \quad h_0 = -8.0\,\text{eV}, \; \alpha = 1.5\,\text{Å}^{-1}, \; r_0 = 1.5\,\text{Å}, \tag{3}$$

$$\Pi_F^{3F} = \sum_{(i,j,k) \in F} \cos\theta_{ijk} \, (\varepsilon_i + \varepsilon_j + \varepsilon_k)/3, \tag{4}$$

$$\Pi_F^{4F} = \sum_{(i,j,k,l) \in F} \cos(2\phi_{ijkl}) \, (\varepsilon_i + \varepsilon_j + \varepsilon_k + \varepsilon_l)/4. \tag{5}$$

The values $h_0 = -8.0\,\text{eV}$, $\alpha = 1.5\,\text{Å}^{-1}$, $r_0 = 1.5\,\text{Å}$ are typical Wolfsberg–Helmholz constants for second-period σ bonds and are held fixed across all benchmarks reported here. Because each figure class carries its own linear coefficient J_F, a global mismatch in the prefactor $h(r)$ for a given element pair is absorbed by ridge regression and does not bias inter-molecular ranking.

1.5 Per-atom features and ridge regression

For each molecule we compute the figure totals Π_F and divide by the total atom count, yielding intensive per-atom features. The regression target is the per-atom *formation* energy $y = E_{\mathrm{DFT}}^{\mathrm{form}}/N_{\mathrm{atoms}}$, i.e. the SPICE DFT energy with the atomic self-energies subtracted (Section S2); total-energy results are tabulated alongside in Section S4 for reference. Ridge regression solves

$$\min_{J,\,b} \sum_{i} \left(y_i - J^{\top}\Pi_i - b\right)^2 + \alpha \, \|J\|_2^2, \tag{6}$$

with α optimised by 5-fold cross-validation over a logarithmic grid in $[10^{-4},\, 10^{2}]$. Parent-stratified splits use a fixed random seed (42) and a 20% test fraction throughout.

1.6 Leading coefficients of the formation-energy fit

Table 1 lists the twelve features with the largest standardised ridge coefficients $|J_F|$ (features standardised on the training set) for the dipeptide formation-energy model. The dominant terms are all $2F$ bonding eigenvalues of the bonds that define peptide energetics – the amide C–N, the carbonyl C=O, and the C–H and C–C bonds – confirming that, once atomic self-energies are removed, the model's weight is carried by bond-formation physics rather than by composition.

rank	feature (figure, atoms, bond order)	J_F (std.)
1	$2F$ C(p)–N(p), single (amide)	+1.07
2	$2F$ C(p)–O(p), double (carbonyl)	+0.90
3	$2F$ C(s)–H(s), single	−0.87
4	$2F$ C(p)–H(s), single	+0.74
5	$2F$ C(s)–N(s), single	−0.70
6	$2F$ C(p)–C(s), single	−0.69
7	$2F$ C(s)–N(p), single	−0.64
8	$2F$ C(p)–O(s), double	−0.60
9	$2F$ H(s)–N(s), single	−0.53
10	$2F$ H(s)–N(p), single	+0.45
11	$2F$ C(s)–O(s), double	−0.40
12	$2F$ C(p)–N(p), double	+0.38

Table 1: Twelve largest standardised ridge coefficients of the dipeptide formation-energy model (1F+2F+3F basis). All are $2F$ bonding eigenvalues of peptide-relevant bonds. Reproducible via `results/spice/formation_energy_test.py`.

2 SPICE 2.0 dataset access

The SPICE 2.0.1 release was downloaded from `https://doi.org/10.5281/zenodo.10975225` as a single 37.5 GB HDF5 file. We extracted three subsets, each as a local JSON cache:

subset	molecules	conformers/parent (max)	total samples
SPICE Dipeptides v1.3	677	5	3 385
SPICE PubChem Set 1 v1.3	500	5	2 500
SPICE DES370K v1.0	500	10	4 740

Both the per-conformer DFT total energy and the SPICE formation energy (total en-

ergy minus the tabulated atomic self-energies, all at the ωB97M-D3BJ/def2-TZVPPD level) are stored in the HDF5 file. We use formation energies as the primary regression target (Section S1.5).

3 Per-parent error tables

The eight worst-fitted dipeptide parents (highest mean absolute error in the parent-stratified formation-energy test set) are listed below; full per-parent tables are deposited at the Zenodo archive. The worst cases are dominated by amino-acid pairs with strong inter-residue electrostatics, consistent with the absence of an explicit long-range term in the covalent basis (Section S7). The full distribution of within-parent Spearman correlations is shown in the main text (Figure 3).

parent	MAE [meV/atom]	σ [meV/atom]
asp-arg	99.2	9.2
arg-cyx	98.4	33.8
asp-hip	83.9	18.8
lys-glu	76.9	12.2
hip-ala	63.0	18.8
ash-hip	58.8	22.6
hip-gly	58.5	10.1
pro-hip	58.4	15.1

4 Total- versus formation-energy targets

Total per-atom energies are dominated by element-dependent atomic self-energies: their spread across distinct molecules is enormous (778 eV per atom on dipeptides), so a small per-atom RMSE trivially yields Spearman $\rho \approx 1$ and the ranking reduces to a composition count. We therefore adopt *formation* energies as the primary target, where the self-energies

are removed and the target spread is only a fraction of an eV per atom. Both fits use the identical 414-feature ($1F + 2F + 3F$) basis, the identical parent-stratified split, and ridge regression (Table 2).

subset	target	RMSE [meV/atom]	inter ρ	R^2	target spread [eV/atom]
Dipeptides	total	26.8	0.9999	1.000	778.5
	**formation**	**30.5**	**0.973**	**0.949**	**0.646**
PubChem-1	total	157.8	0.9999	1.000	14 499.6
	**formation**	**89.3**	**0.980**	**0.913**	**2.084**
DES370K	total	632.3	0.9999	1.000	43 172.2
	**formation**	**376.4**	**0.982**	**0.628**	**4.178**

Table 2: Total- vs formation-energy fits (identical basis, split and solver). On formation energies the inter-molecular ranking remains strong while no longer being driven by composition: on dipeptides the model resolves 95% of the variance of a target whose test spread is only 0.13 eV per atom. On PubChem the formation-energy RMSE is actually *lower* than the total-energy RMSE, because the regression no longer has to absorb the large self-energy offsets. DES370K retains the lowest R^2, consistent with its ion-pair content and the absence of a long-range term (Section S7). Reproducible via `results/spice/formation_energy_test.py`.

5 Head-to-head with MACE-OFF23 on the identical test set

We evaluated the public MACE-OFF23 checkpoints (small, medium, large; `mace-torch` 0.3.15)[3] on the *identical* parent-stratified dipeptide test set used for OCE (680 conformers, 136 held-out parents). Two methodological points are essential for a fair comparison. (i) MACE-OFF23's headline SPICE energy RMSE ($\sim$1 meV per atom) is measured on a random *conformer* split, an interpolative task; our parent-stratified split holds out entire molecules

and is substantially harder. (ii) A large fraction of the dipeptides carry a *net molecular charge* (protonated Arg/Lys/His$^+$; deprotonated Asp/Glu$^-$). MACE-OFF (and the covalent OCE basis alike) receives no total-charge input and therefore models these as neutral, which is the single largest error source for both methods. We split the test set accordingly and remove a per-element atomic-reference offset from the MACE energies by least squares (the convention difference between MACE-OFF and SPICE-formation references); the residual is the genuine relative-energy error (Table 3).

	RMSE [meV/atom]		
method	neutral	charged	intra ρ
MACE-OFF23 small	34.7	60.7	0.956
MACE-OFF23 medium	34.1	59.8	0.962
MACE-OFF23 large	34.1	59.5	0.965
OCE (this work)	**25.5**	**40.3**	0.088

Table 3: MACE-OFF23 versus OCE on the identical held-out dipeptide test set, split by net molecular charge (E0-aligned per-atom RMSE). Two conclusions are robust. (1) The *intra-conformer* Spearman cleanly separates the methods: MACE-OFF resolves conformers ($\rho \approx 0.96$) while OCE cannot ($\rho \approx 0.09$) – the dual regime of the main text, now quantified against the state-of-the-art competitor and unaffected by any reference or charge confound (composition and charge are fixed within a parent). (2) On this strict molecule-holdout split, OCE's relative-energy RMSE is of the same order as MACE-OFF's, and the charged species are the dominant error source for *both* methods, underscoring that the missing-electrostatics limitation is shared rather than specific to the linear basis. We do not read this as OCE being more accurate than MACE-OFF in general – MACE is fit to a global loss over broad SPICE chemistries and resolves geometry, which OCE does not; the comparison is offered only to place the two on the same footing for this task. Reproducible via `results/spice/mace_off_diagnose.py` and `neutral_charged_split.py`.

6 Cross-subset transferability

To test prediction on molecule classes absent from training, we trained the ridge model on all of one subset and predicted all of another, on formation energies, over a shared union feature space (2 860 columns; Table 4).

train → test	RMSE [meV/atom]	ρ	R^2
PubChem-1 → Dipeptides	47.1	0.927	0.865
PubChem-1 → DES370K	272.6	0.913	0.751
Dipeptides → PubChem-1	320.4	0.674	−0.225
Dipeptides → DES370K	816.8	0.334	−1.236
DES370K → Dipeptides	172.8	0.498	−0.824
DES370K → PubChem-1	442.6	0.526	−1.337

Table 4: Cross-subset transfer of formation-energy predictions. A chemically broad training set (PubChem, 10 elements) transfers to entirely unseen classes – peptides ($\rho = 0.93$, $R^2 = 0.87$) and non-covalent dimers ($\rho = 0.91$, $R^2 = 0.75$) – whereas narrow training sets extrapolate poorly. This is the expected behaviour of a linear model: it predicts within the span of figures present in training and cannot synthesise coefficients for unseen figure types, a transparent and checkable condition. Reproducible via `results/spice/transfer_and_charge_test.py`.

7 Long-range electrostatics: Coulomb / Madelung feature

The localised LCAO σ-bonding form used for Π^{2F} decays exponentially and cannot represent long-range Coulomb interactions, which dominate the ion-pair dimers in DES370K. The baseline basis was kept deliberately local and covalent to test the cleanest possible hypothesis – how far a connectivity-only expansion can reach – and the cost of that choice is reported transparently: DES370K has the lowest R^2 and its worst-fit cases are exactly the monopolar ion pairs. Charges are, however, straightforward to include. We append a single explicit

Coulomb feature

$$\Pi_{\mathrm{M}} = \frac{1}{N_{\mathrm{atoms}}} \sum_{i<j,\, r_{ij}>r_{\min}} \frac{k_C\, q_i\, q_j}{r_{ij}}, \qquad k_C = 14.3996\,\mathrm{eV}\cdot\text{Å}, \tag{7}$$

with q_i the formal SMILES charges aligned to the SPICE atom-map labels (deliberately *not* DFT-derived, to avoid circularity; 252 of 500 DES370K parents are ionic, the rest neutral) and $r_{\min} = 1.5$ Å excluding covalent pairs already in the $2F$ basis.

On the original *total*-energy target this single term lowered the DES370K RMSE from 588 to 488 meV per atom, with the largest gain on the neutral sub-block (335 → 85 meV per atom, −75%) and a fitted coefficient $J_{\mathrm{M}} = 0.84$ close to the physical value of unity. Repeating the ablation on the *formation*-energy target (the new primary metric) confirms the same conclusion (Table 5): a single additive linear term improves the fit, with the gain concentrated on the charged dimers, as physics demands.

DES370K test set (formation E)	RMSE [meV/atom]	R^2
all dimers ($n = 943$), baseline $1F$+$2F$+$3F$	376.4	0.628
all dimers ($n = 943$), + Coulomb	365.3	0.650
charged ion-pairs only ($n = 503$), baseline	510.3	0.502
charged ion-pairs only ($n = 503$), + Coulomb	495.1	0.531

Table 5: Effect of one formal-charge Coulomb feature on DES370K formation-energy fits. The improvement concentrates on the charged subset. A screened-Coulomb / Ewald 2-figure and MBIS partial charges are the natural extensions for charged and periodic systems.

8 Ablation: 3F versus 4F basis

basis	features	inter ρ (form.)	intra ρ
$1F + 2F + 3F$ (Dipeptides)	414	0.973	0.088
$1F + 2F + 3F + 4F$ (Dipeptides)	886	0.974	0.080

Adding the dihedral basis $4F$ reduces the formation-energy RMSE only marginally and does not change the conformational regime (intra ρ stays near zero), supporting the structural argument in the main text that conformational variation is invisible to a graph-adjacency basis.

9 Hyperparameter sensitivity

Ridge regularisation was selected by 5-fold cross-validation over $\alpha \in \{10^{-4}, 10^{-3.5}, \ldots, 10^{2}\}$. The optimal α fell in 10^{-3}–10^{0} across subsets and targets. Sensitivity to this choice is below 5 meV per atom in test-set RMSE.

10 Computational environment

Python 3.10. Dependencies: ASE 3.22.1,[4] scikit-learn 1.4,[5] h5py 3.10, NumPy 1.26, SciPy 1.11. xtb 6.6.1[1] single-atom calculations generated the orbital reference tables; the MACE-OFF23 evaluation (Section S5) used `mace-torch` 0.3.15. All OCE benchmarks were performed on a single AMD Ryzen 9 7950X workstation; the entire SPICE pipeline (load + features + training + evaluation) for the dipeptide subset completes in under 60 s on a single thread.

11 Code and data availability

Source code is available at `https://github.com/david888azv/OCE-SPICE`. The fitted ridge coefficients, feature caches, per-parent prediction tables, and the revision-1 analysis scripts (`formation_energy_test.py`, `transfer_and_charge_test.py`, `mace_off_headtohead.py`) are deposited at `https://doi.org/10.5281/zenodo.20068240`. Re-running the full pipeline from the original SPICE 2.0.1 HDF5 file requires approximately 10 min of wall-clock time (excluding the one-time HDF5 download).

References

(1) Bannwarth, C.; Ehlert, S.; Grimme, S. GFN2-xTB: an accurate and broadly parametrized self-consistent tight-binding quantum chemical method with multipole electrostatics and density-dependent dispersion contributions. *Journal of Chemical Theory and Computation* **2019**, *15*, 1652–1671.

(2) Cordero, B.; Gómez, V.; Platero-Prats, A. E.; Revés, M.; Echeverría, J.; Cremades, E.; Barragán, F.; Alvarez, S. Covalent radii revisited. *Dalton Transactions* **2008**, *21*, 2832–2838.

(3) Kovács, D. P.; Moore, J. H.; Browning, N. J.; Batatia, I.; Horton, J. T.; Kapil, V.; Witt, W. C.; Magdău, I.-B.; Cole, D. J.; Csányi, G. MACE-OFF: short-range transferable machine learning force fields for organic molecules. *Journal of the American Chemical Society* **2025**, *147*, 17598–17611.

(4) Larsen, A. H.; Mortensen, J. J.; Blomqvist, J.; Castelli, I. E.; Christensen, R.; Dułak, M.; Friis, J.; Groves, M. N.; Hammer, B.; Hargus, C. et al. The atomic simulation environment – a Python library for working with atoms. *Journal of Physics: Condensed Matter* **2017**, *29*, 273002.

(5) Pedregosa, F.; Varoquaux, G.; Gramfort, A.; Michel, V.; Thirion, B.; Grisel, O.; Blondel, M.; Prettenhofer, P.; Weiss, R.; Dubourg, V. et al. Scikit-learn: machine learning in Python. *Journal of Machine Learning Research* **2011**, *12*, 2825–2830.
